# Induction of Pro-Apoptotic Endoplasmic Reticulum Stress in Multiple Myeloma Cells by NEO214, Perillyl Alcohol Conjugated to Rolipram

**DOI:** 10.3390/ijms19010277

**Published:** 2018-01-17

**Authors:** Thomas C. Chen, Nymph Chan, Shirin Labib, Jiali Yu, Hee-Yeon Cho, Florence M. Hofman, Axel H. Schönthal

**Affiliations:** 1 Department of Neurological Surgery, Keck School of Medicine, University of Southern California, Los Angeles, CA 90089, USA; nymphcha@usc.edu (N.C.); jyu28@vols.utk.edu (J.Y.); heeyeonc@usc.edu (H.-Y.C.); 2 Department of Molecular Microbiology and Immunology, Keck School of Medicine, University of Southern California, Los Angeles, CA 90089, USA; shi.labib@gmail.com (S.L.); 3 Department of Pathology, Keck School of Medicine, University of Southern California, Los Angeles, CA 90089, USA; hofman@usc.edu

**Keywords:** bortezomib, CHOP, perillyl alcohol, phosphodiesterase, rolipram

## Abstract

Despite the introduction of new therapies for multiple myeloma (MM), many patients are still dying from this disease and novel treatments are urgently needed. We have designed a novel hybrid molecule, called NEO214, that was generated by covalent conjugation of the natural monoterpene perillyl alcohol (POH), an inducer of endoplasmic reticulum (ER) stress, to rolipram (Rp), an inhibitor of phosphodiesterase-4 (PDE4). Its potential anticancer effects were investigated in a panel of MM cell lines. We found that NEO214 effectively killed MM cells in vitro with a potency that was over an order of magnitude stronger than that of its individual components, either alone or in combination. The cytotoxic mechanism of NEO214 involved severe ER stress and prolonged induction of CCAAT/enhancer-binding protein homologous protein (CHOP), a key pro-apoptotic component of the ER stress response. These effects were prevented by salubrinal, a pharmacologic inhibitor of ER stress, and by *CHOP* gene knockout. Conversely, combination of NEO214 with bortezomib, a drug in clinical use for patients with MM, resulted in synergistic enhancement of MM cell death. Combination with the adenylate cyclase stimulant forskolin did not enhance NEO214 impact, indicating that cyclic adenosine 3′,5′-monophosphate (AMP) pathways might play a lesser role. Our study introduces the novel agent NEO214 as a potent inducer of ER stress with significant anti-MM activity in vitro. It should be further investigated as a potential MM therapy aimed at exploiting this tumor’s distinct sensitivity to ER stress.

## 1. Introduction

Among the various tasks of the endoplasmic reticulum (ER) is proper protein folding and processing. A variety of physiological and pathophysiological conditions, as well as a number of pharmacological agents, are able to disturb this process, thereby causing ER stress. The cell reacts to ER stress by initiating a defensive process, called the unfolded protein response (UPR), which pursues two objectives: first, in the case of moderate stress, the defensive module of this system attempts to neutralize or adapt to the insult and restore proper homeostasis. Second, in the case of excessively aggravated stress, the UPR switches to its pro-apoptotic module and initiates apoptosis [1]. Among the executor proteins of the apoptotic component is CHOP (CCAAT/enhancer binding protein homologous transcription factor, also called GADD153), a transcription factor that alters the transcriptional profile of cells and triggers a pro-apoptotic program [2]. Prolonged expression of CHOP is a key trigger for apoptosis, and therefore this protein is commonly used as a marker to indicate that pro-apoptotic events of the UPR have been initiated [3].

Chronically elevated levels of ER stress have been implicated in a number of diseases, and efforts are underway to develop approaches to target ER stress mechanisms for therapy. For example, obesity and type 2 diabetes have been linked to ER stress-induced failure of insulin-producing pancreatic beta cells, and current research efforts are aimed at developing drugs that ameliorate cellular stress and thereby protect beta cell function [4,5]. The opposite approach is being investigated in the context of cancer therapy. Here, research efforts aim at further aggravating pre-existing ER stress, seeking to reach the pro-apoptotic threshold to initiate cell death [6]. Secretory cancer cells, such as multiple myeloma (MM), are attractive targets for this approach, because in addition to the general demands of proliferating cancer cells, these cells highly depend on proper ER functioning for their extensive immunoglobulin production [7].

Treatment options for MM have increased over the past 10 years and have resulted in improved survival rates among MM patients. However, there is a high relapse rate, and the disease remains incurable in many cases [8]. Bortezomib, the first of several clinically used proteasome inhibitors, is used across the entire spectrum of myeloma disease, either as single agent or combined with other therapies [9]. Inhibition of proteasome function by bortezomib results in the accumulation of normal proteins that were bound for destruction and of misfolded, unfolded, and otherwise damaged proteins. This accumulation of superfluous proteins triggers ER stress and causes effective stimulation of the pro-apoptotic UPR module, in particular prolonged expression of CHOP, resulting in proteotoxic cell death [10,11]. In this context, ER stress has been considered the Achilles’ heel of MM [12].

We have been studying approaches to further aggravate chronic ER stress in cancer cells in order to achieve optimized tumor cell killing. We showed previously that certain combinations of ER stress-aggravators (ERSAs), i.e., pharmacological agents that aggravate low-level, chronic ER stress that exists in tumor cells, result in superior anticancer outcomes in vitro and in vivo [6,13]. For example, we demonstrated that the combination of bortezomib with the cyclooxygenase-2 (COX2) inhibitor celecoxib potently aggravated ER stress and resulted in strongly enhanced tumor cell death [14]. Others have shown potent combination effects of bortezomib and the HIV-1 protease inhibitor nelfinavir, which also is known to trigger pro-apoptotic ER stress [15,16]. In the current study, we have further explored the principle of aggravating ER stress and have created a novel hybrid molecule with potent ERSA characteristics. This molecule, called NEO214, consists of two pharmacologically distinct compounds, rolipram (Rp) and perillyl alcohol (POH).

Rp is a selective phosphodiesterase-4 (PDE4) inhibitor that was in clinical development as an anti-depressant in the early 1990s. However, clinical trials revealed that it required fairly high dosing that resulted in the emergence of significant side effects, primarily dose-limiting gastrointestinal (GI) toxicities, and therefore it was abandoned [17]. More recently, efforts have started to repurpose Rp for cancer therapy and other applications. For example, it was demonstrated in vitro that Rp could inhibit proliferation of cancer cell lines derived from different cancer types [18,19,20,21,22]. In vivo, Rp was shown to slow intracranial glioblastoma growth when added to first-line therapy consisting of temozolomide and radiation [23]. Among the various non-cancer applications being explored for Rp is its neuroprotective function, which has shown significant benefit in mouse models of traumatic spinal cord injury [24].

Perillyl alcohol (POH) is a monoterpene and a natural constituent of lavender and lilac oil, cherries, cranberries, spearmint, and certain other plants [25]. Although this compound had shown promising activity in several preclinical cancer models, it did not enter clinical practice, primarily because dose-limiting GI toxicity became evident in clinical trials with oral POH [26]. However, recent phase I/II clinical studies in Brazil indicated that simple intranasal inhalation of POH was effective against recurrent glioblastoma, in the absence of detectable toxic events [27], and similar clinical studies have started in the United States (NCT02704858).

The molecular mechanism of action of POH is not entirely clear. Initially, it was characterized as an inhibitor of Ras oncoprotein [28,29], but a number of experimental models have challenged this view [30,31,32]. Additional identified POH targets include *c-fos* and *c-jun* proto-oncogenes [33], transforming growth factor beta (TGFβ) receptor [34], nuclear factor kappa B (NF-κB) [35], mammalian target of rapamycin (mTOR) [36], components of the cell cycle machinery [37,38], and certain cellular enzymes, such as telomerase [39,40] and sodium/potassium adenosine triphosphatase (Na/K-ATPase) [41]. Altogether, it is likely that POH exerts pleiotropic impact on several of these targets simultaneously, and at least some of them result in aggravated ER stress [42], cell cycle arrest [34] and/or induction of apoptosis [43] (see further details and additional references in recent review: [26]).

We have created a novel molecule, NEO214, where POH was covalently conjugated to Rp. Initially, the idea behind this approach was to devise a chimeric chemotherapeutic agent that potentially might be suitable for intranasal delivery, thus avoiding the limitations due to GI toxicity after oral delivery. Here we report on the in vitro characterization of NEO214’s biological activity and ability to trigger tumor cell death and present evidence of its molecular mechanism of action, which involves potent ERSA activity.

## 2. Results

### 2.1. Cytotoxic Potency of NEO214 is Greater Than the Sum of Its Parts

**Figure 1:**
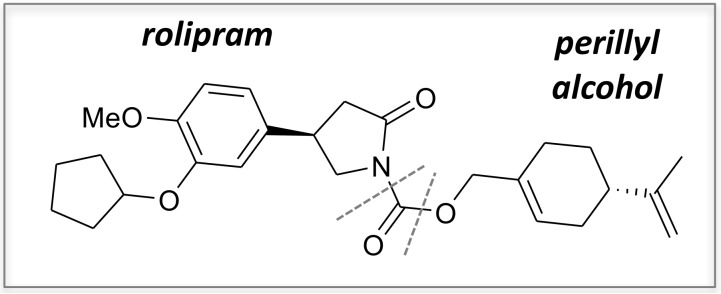
Chemical structure of NEO214. NEO214 was generated by covalently linking rolipram to perillyl alcohol via a carbamate bridge (shown between dotted lines).

**Figure 2:**
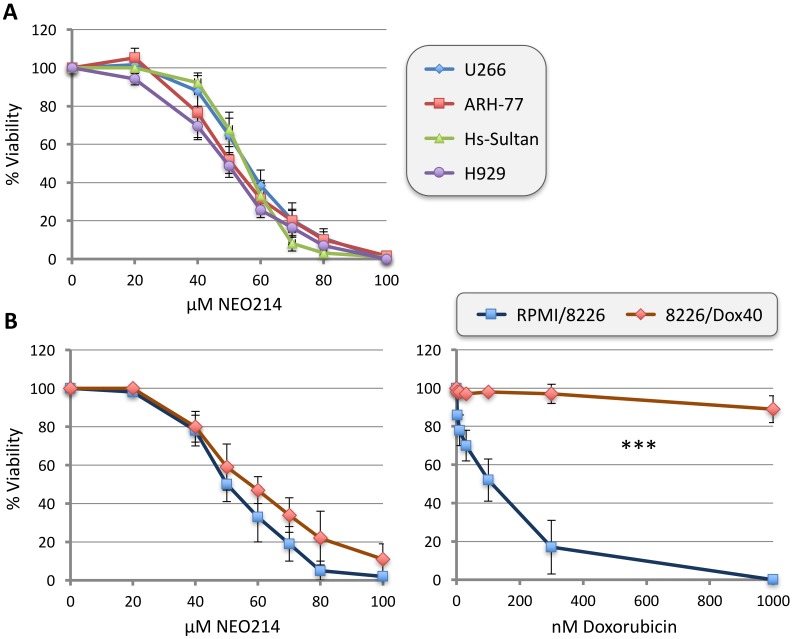
NEO214 kills multiple myeloma (MM) cells in vitro. Different MM cell lines were treated at increasing concentrations of NEO214, and cell viability was determined 48 h later by standard Methylthiazoletetrazolium (MTT) assay. (**A**) U266, ARH-77, H929, and Hs-Sultan cells were used. (**B**) RPMI/8222 and 8226/Dox40 cells were used. The right panel demonstrates very large differential in response to doxorubicin treatment, confirming high drug-resistance status of the Dox40 subline. Asterisks (***): statistical difference between data points ≥ 25 nM doxorubicin: *p* < 0.001). In all cases, vehicle-only treated cells, as well as entirely untreated cells, were used as controls (there was no difference between the two). Survival of untreated cells was set at 100%. Data points are mean ± SE from ≥3 independent experiments.

Rp and POH were covalently conjugated via a carbamate bridge to create the novel chemical entity NEO214 (Figure 1). The cytotoxic in vitro activity of NEO214 was studied in five different human MM cell lines, RPMI/8226, U266, H929, ARH-77, and Hs-Sultan. Cells were incubated with increasing concentrations of NEO214 for 48 h, and cell viability was determined by standard Methylthiazoletetrazolium (MTT) assay. As presented in Figure 2A, all cells were similarly sensitive to killing by NEO214, with an IC50 of about 50 µM. Importantly, 8226/Dox40 cells, a multidrug-resistant variant of the RPMI/8226 cells, were sensitive to NEO214 as well (Figure 2B). Repetitions of all MTT assays at different cell densities and different incubation times yielded similar outcomes.

**Figure 3:**
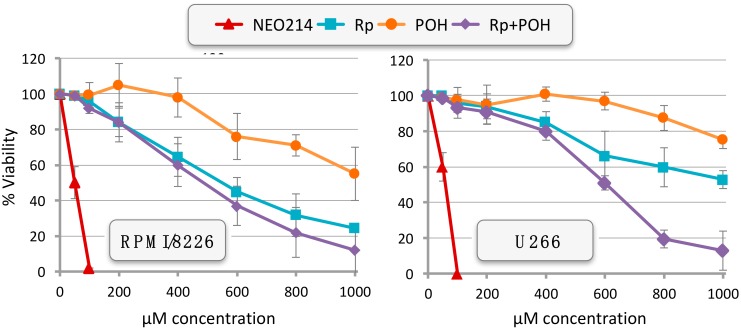
NEO214 is more cytotoxic than the sum of its parts. RPMI/8226 and U266 cells were treated with increasing concentrations of NEO214, Rolipram (Rp), perillyl alcohol (POH), or an equimolar mix of Rp plus POH. Cell survival was determined 48 h later by MTT assay. Survival of vehicle-only treated cells was set at 100%. Data points are mean ± SE from ≥3 independent experiments.

We next compared these effects to those of the individual constituents of NEO214, Rp and POH, either alone or in combination. As shown in Figure 3 with RPMI/8226 and U266 cells, the application of Rp alone or POH alone required 10–20 times higher concentrations in order to reach the cytotoxic IC50. Intriguingly, an equimolar mix, consisting of Rp and POH as individual, non-conjugated compounds, also was unable to mimic the strong cytotoxic potency of the conjugated compound. Table 1 summarizes these comparisons for all 5 MM cell lines and the drug-resistant 8226/Dox40 line. As shown, the average cytotoxic IC50 values were in the range of 50–59 µM for NEO214, whereas the values for Rp ranged from 551 µM to over 1 mM, and those of POH all were above 1 mM. Equimolar mixes of Rp plus POH as individual compounds yielded IC50s from 482–789 µM, demonstrating that NEO214’s potency was an order of magnitude greater than the potency of the sum of its parts.

In comparison to these cancer cell lines, we also determined the effects of NEO214 on non-tumor cells from other tissues, i.e., immortalized breast epithelial cells, primary astrocytes, and primary brain endothelial cells. As displayed in Table 1, in the case of immortalized cells, the IC50 of NEO214 approximately doubled, to an average of 118 µM. In the case of truly normal (i.e., non-immortalized) primary cells, there was no detectable toxicity even at the highest concentrations of NEO214 used, which was 200 µM. Thus, there was a clear, statistically significant (*p* < 0.01) difference in NEO214 sensitivity between the above MM cell lines and the tested non-tumor cells, suggesting that NEO214 perhaps could be clinically useful.

### 2.2. NEO214 Inhibits Phosphodiesterase 4

**Figure 4:**
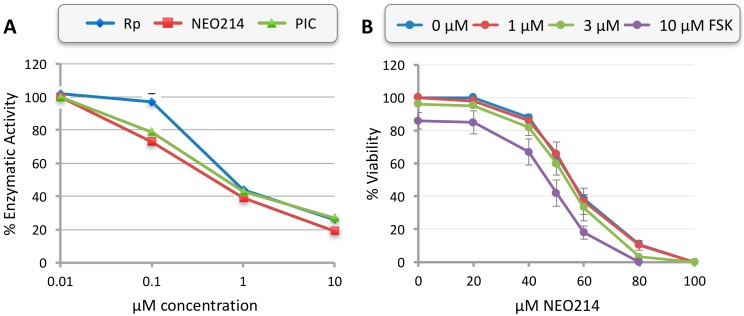
In vitro PDE4 activity and combination with forskolin. (**A**) The enzymatic activity of purified, recombinant PDE4A1A was determined in the presence of increasing concentrations of rolipram (Rp), NEO214, or piclamilast (PIC). Data shown are averages from two independent repeats, each one performed in duplicate. (**B**) RPMI/8226 cells were treated with combinations of NEO214 and forskolin (FSK). After 48 h, cell viability was determined by MTT assay. Survival of vehicle-only treated cells was set at 100%. Data points are mean ± SE from two experiments with each data point in triplicate.

To gain insight into the mechanisms of NEO214-induced cell death, we focused on the two processes that were known to be targeted by its individual components, i.e., inhibition of PDE4 enzymatic activity by Rp and aggravation of ER stress by POH. First, the in vitro activity of purified, recombinant PDE4 was determined in the presence of NEO214, Rp, or piclamilast (a PDE4-specific inhibitor). As shown in Figure 4A, all three compounds demonstrated potent PDE4-inhibitory activity at very similar IC50s in the sub-micromolar range. It therefore appeared that Rp’s inhibitory potency was faithfully retained within the NEO214 molecule.

As an approach to determine whether inhibition of PDE4 might be critical to mediating NEO214’s cytotoxic impact, we treated cells with NEO214 in the presence or absence of forskolin (FSK). Our rationale was that stimulation of adenylate cyclase activity with FSK should greatly enhance any potential cytotoxic effects initiated by inhibition of PDE4 with NEO214, due to more effective accumulation of cyclic AMP. However, the results presented in Figure 4B do not appear to support this view. We found that the addition of FSK did not substantially increase overall cytotoxic impact over that which would be expected from the sum of these two compounds. FSK at 1 or 3 µM was unable to increase cytotoxic activity of NEO214 alone; FSK at 10 µM exerted some toxicity by itself (~15% reduction of viability) and in combination with NEO214 enhanced overall toxicity by approximately this margin (Figure 4B). Therefore, these results did not point to a major role of cyclic AMP signaling or PDE4 inhibition in mediating NEO214’s anticancer effects.

### 2.3. NEO214 Triggers Pro-Apoptotic ER Stress

To gain further insight into the mechanisms of NEO214-induced cell death, we next investigated several established markers as readouts of specific cellular processes. The expression level of activating transcription factor 3 (ATF3) was used as a general cell stress indicator. CHOP was used as the well-characterized marker of activation of the pro-apoptotic module of the ER stress response/UPR. In addition, we analyzed typical markers of apoptosis, specifically the proteolytic cleavage of PARP (poly-ADP-ribose polymerase), the activation of caspase 7 (C-7), and emergence of phosphorylated HA2X protein (γ-HA2X, indicating damage and degradation of DNA).

**Figure 5:**
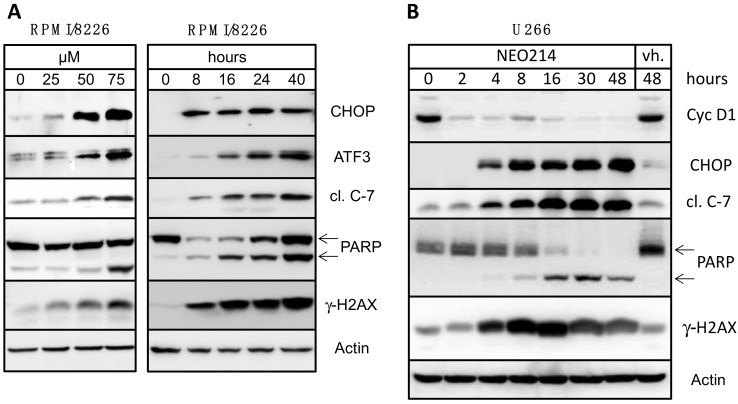
NEO214 induces markers of ER stress and apoptosis. (**A**) RPMI/8226 cells were treated with increasing concentrations of NEO214 for 24 h (left panel) or with 100 µM NEO214 for different times (right panel). (**B**) U266 cells were treated with 100 µM NEO214 for different times, and with vehicle only (vh.) for 48 h. In all cases, cell lysates were prepared and subjected to Western blot analysis with specific antibodies as indicated. Actin was used as the loading control. cl. C-7: cleaved caspase 7 (band represents active fragment). Two arrows point to full-length and cleaved form of poly-ADP-ribose polymerase (PARP). CHOP, CCAAT/enhancer-binding protein homologous protein; ATF3, activating transcription factor 3, γH2AX, phosphorylated form of histone variant H2AX protein.

RPMI/8226 cells were treated with different concentrations of NEO214 and for different time points. Figure 5A shows that NEO214 triggered pronounced activation of all these markers, signifying severe cellular stress and ensuing apoptosis. Importantly, increased levels of CHOP could be detected fairly early, at 8 h, and did not diminish even at 40 h, indicating that the pro-apoptotic cellular response was maintained and could not be subdued by the pro-survival/adaptive module of the UPR. This observation was further supported by continuously increasing levels of apoptotic markers, activated caspase 7, cleaved PARP, and γ-HA2X. Nearly identical results were also obtained with U266 cells, confirming the observations in a different cell line (Figure 5B).

Because increased CHOP expression indicated severe ER stress, we also determined expression levels of cyclin D1, a cell cycle protein essential for cell proliferation. A well characterized feature of ER stress is inhibition of cellular protein synthesis (with select exception of stress proteins such as ATF3, CHOP and others), and therefore short-lived proteins like cyclins will quickly disappear from the cell’s protein inventory. This was indeed the case in response to treatment with NEO214: treatment of U266 cells with NEO214 resulted in rapid disappearance of cyclin D1 protein (Figure 5B), further revealing the presence of drug-induced ER stress. Notably, cyclin D1 expression did not recover over 48 h, showing that the pro-survival module of the ER stress response was unable to subdue its pro-apoptotic, CHOP-controlled component.

**Figure 6:**
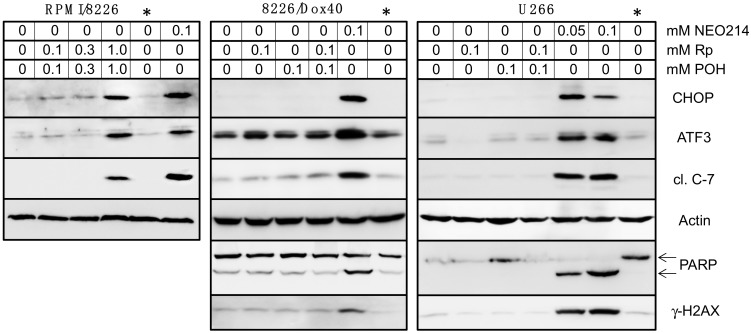
NEO214 is more potent than the sum of its parts. RPMI/8226, 8226/Dox40, and U266 cells were treated with Rp alone, POH alone, and Rp plus POH in combination, at the indicated concentrations. For comparison purposes, cells were also treated with NEO214 in parallel. Control cells remained untreated (left lane in each panel) or were treated with vehicle (lanes marked by asterisk: *). After 24 h, cell lysates were prepared and subjected to Western blot analysis with actin as the loading control. Note effective induction of stress markers (CHOP and ATF3) and apoptosis markers (cleaved/active caspase 7, PARP and γ-HA2X) by 0.05 and 0.1 mM NEO214, whereas Rp and POH, alone or in combination, did not affect expression of these markers, unless their concentrations were increased to 1 mM each (left panel). (Due to the high concentrations included, drug concentrations in this figure are shown in units of millimolar).

As our cytotoxicity assays had shown that the individual components of NEO214—Rp and POH—were unable to mimic the strong cytotoxic potency of the conjugated compound, we next investigated whether this would also hold true with regard to the above protein markers of stress and apoptosis. RPMI/8226, 8226/Dox40, and U266 cells were treated with Rp alone, POH alone, or Rp combined with POH at equimolar concentrations, and the readouts were compared to those of NEO214 treatment. When used at 100 µM, treatment of these cells with Rp, POH, or Rp plus POH did not result in any detectable increase of the investigated stress markers or any of the apoptotic indicators (Figure 6). In comparison, NEO214 at the same concentration triggered a strong response, including in multidrug-resistant 8226/Dox40 cells. Further increased concentrations for combination treatments, where 300 µM Rp was mixed with 300 µM POH, also did not result in an appreciable response. Only when 1000 µM Rp was combined with 1000 µM POH was an effect observed (Figure 6, left panel). Thus, with regard to the prominent induction of stress markers and apoptotic indicators, NEO214 was about 10–20-fold more potent than the sum of its parts, i.e., the combination of Rp plus POH.

### 2.4. ER Stress and CHOP are Key to NEO214-Induced Cell Death

**Figure 7:**
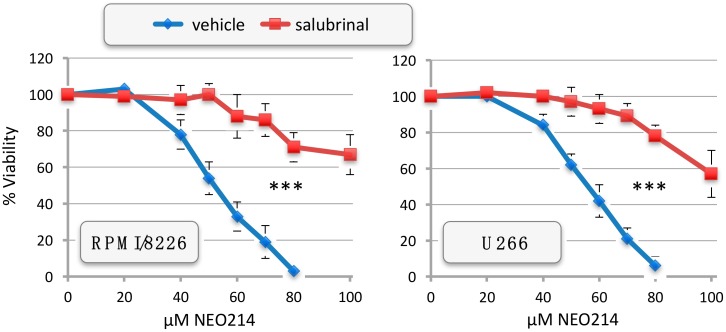
Salubrinal antagonizes NEO214 toxicity. RPMI/8226 and U266 cells were treated with increasing concentrations of NEO214 in the presence or absence of 75 µM salubrinal. After 48 h, cell viability was determined by standard MTT assay. Similar results were obtained at 24 h and at 72 h. Asterisks (***): *p* < 0.001 between salubrinal and vehicle at NEO214 concentrations ≥ 50 µM.

**Figure 8:**
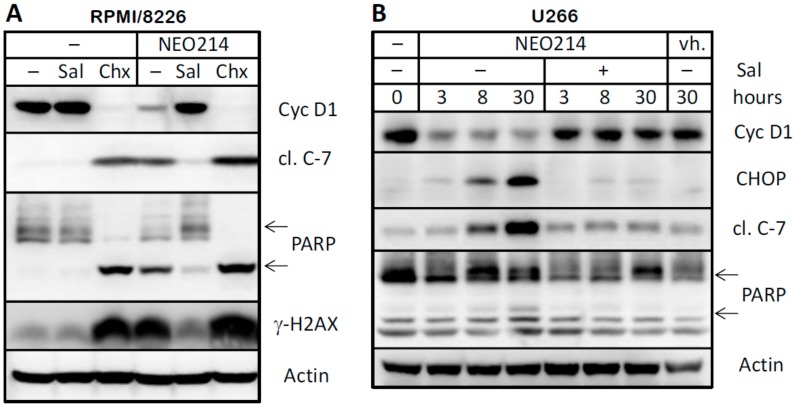
Salubrinal prevents NEO214-induced ER stress and apoptosis. (**A**) RPMI/8226 cells were treated with or without 100 µM NEO214 in the presence or absence of 75 µM salubrinal (Sal) for 24 h. In parallel, 50 µM cycloheximide was included as a positive control for inhibition of protein synthesis and resulting apoptosis. (**B**) U266 cells were treated with or without NEO214 in the presence or absence of salubrinal for different times. In all cases, lysates were prepared and subjected to Western blot analysis with specific antibodies as indicated. Actin was used as the loading control. Cyc D1: cyclin D1; cl. C-7: cleaved/activated caspase 7. Two arrows point to full-length and cleaved form of PARP. vh.: vehicle.

We next investigated the extent by which NEO214-induced ER stress contributed to ensuing cell death. We used salubrinal, an established inhibitor of cellular ER stress processes. MM cells were treated with NEO214 in the presence or absence of salubrinal, and cell viability was determined by MTT assay. As presented in Figure 7, salubrinal was able to potently blunt the toxic impact of NEO214 in both RPMI/8226 and U266 cells, as indicated by significantly (*p* < 0.001) increased cell survival in the presence of this ER stress inhibitor. Salubrinal’s protective effect could be validated at the molecular level as well. As shown in Figure 8, the presence of salubrinal prevented NEO214-induced CHOP expression, activation of caspase 7, cleavage of PARP, H2AX phosphorylation, and downregulation of cyclin D1. Together, these data indicate that NEO214 requires aggravation of ER stress in order to effectively trigger apoptosis.

**Figure 9:**
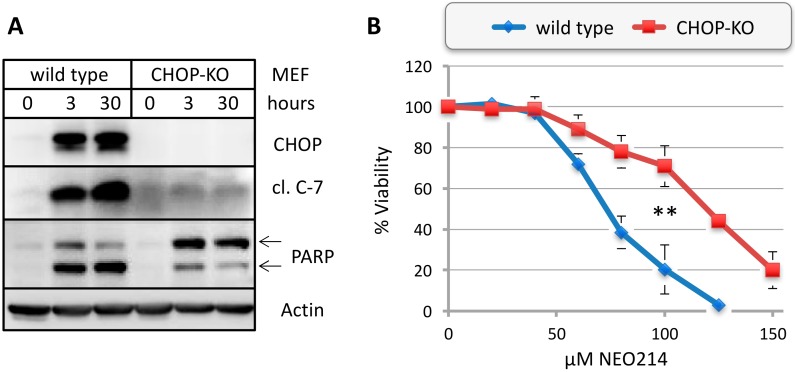
NEO214 requires CHOP for effective cytotoxic impact. Wild type or *CHOP* knockout (*CHOP*-KO) mouse embryo fibroblasts (MEF) were treated with NEO214 and analyzed. (**A**) Cells were treated with 150 µM NEO214 for different times, and cell lysates were analyzed by Western blot. (**B**) Cells were treated with increasing concentrations of NEO214 for 48 h, followed by determination of cell viability by MTT assay. Asterisks (**): *p* < 0.01 between wild type and *CHOP*-KO cells at NEO214 concentrations ≥ 80 µM.

To further investigate the role of CHOP in NEO214-induced cell death, we utilized a pair of well-characterized mouse embryo fibroblasts (MEF), consisting of wild type cells and a *CHOP*-knockout (KO) variant. These cells were exposed to NEO214, followed by the analysis of cell survival by MTT assay and expression levels of key protein markers by Western blot. As shown in Figure 9A, NEO214 treatment of wild type MEF cells resulted in an outcome similar to what was observed in MM cells: there was strong induction of CHOP and potent activation of caspase 7 with pronounced cleavage of PARP. In contrast, *CHOP*-KO cells lacked CHOP induction and potent caspase 7 activation; expression of full-length PARP protein was strongly elevated, but there was less cleavage as compared to wild type cells. At the same time, there was significantly (*p* < 0.01) increased cell survival in *CHOP*-KO cells, although lack of CHOP protein did not completely protect cells from NEO214-induced cell death (Figure 9B). Altogether, these results point to a key role of CHOP in mediating apoptosis induced by NEO214, although other components might contribute as well.

### 2.5. NEO214 Synergizes with Bortezomib

**Figure 10:**
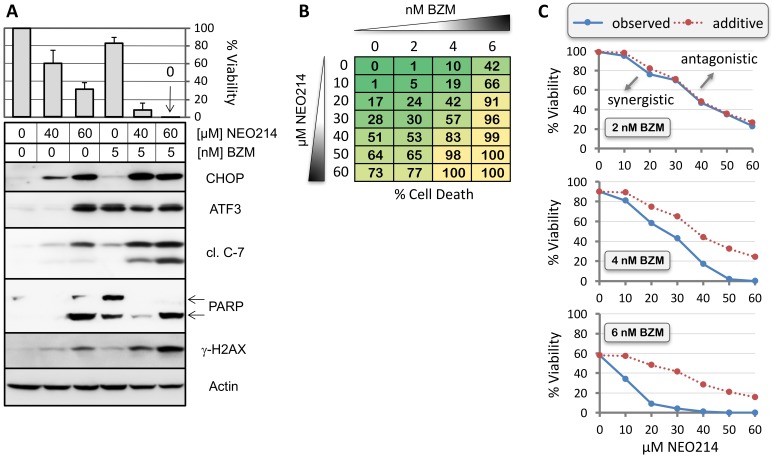
NEO214 synergizes with bortezomib. RPMI/8226 cells were treated with NEO214 alone, bortezomib (BZM) alone, or both in combination. (**A**) Top panel shows cell viability after 48 h of treatment, as determined by MTT assay. Bottom panel shows expression levels of ER stress markers and indicators of apoptosis after 24 h of treatment, as determined by Western blot analysis. Note that the effects of BZM alone on several of these markers were fairly weak, but were substantially enhanced in the presence of NEO214. (**B**) Percent cell death in response to increasing concentrations of BZM (0–6 nM) combined with increasing concentrations of NEO214 (0–60 µM) after 48 h, as determined by MTT assay (averages from six measurements). (**C**) Statistical analysis of drug combination effects of BZM plus NEO214. Dotted red line represents the predicted (i.e., calculated) combination effect (Y_P_) based on the Bliss independence model, which signifies additive effect. Blue straight line shows the observed (i.e., measured) combination response (Y_O_). Overlap of blue line with red line in top graph indicates additive effects at 2 nM BZM. Higher BZM concentrations (4 and 6 nM, middle and bottom panels) result in separation of the lines and pronounced left-shift of blue line, indicating strong synergistic effects. Similar effects were also obtained with U266 cells.

As bortezomib represents the standard of care for MM patients, we next investigated the effects of NEO214 in combination with this proteasome inhibitor. RPMI/8226 cell survival was investigated by MTT assay, and protein markers of cellular stress and apoptosis were determined by Western blot. Figure 10A shows that addition of NEO214 was able to significantly enhance cell death induced by bortezomib. As well, induction of stress markers (ATF3, CHOP) and apoptotic indicators (cleaved caspase 7, PARP and γ-HA2X) was stronger in response to combination treatments as compared to individual treatments with NEO214 or bortezomib. For example, while 5 nM bortezomib or 40 µM NEO214 individually triggered only weak induction of these markers, their combination resulted in much more pronounced effects.

We also determined cell survival during combination treatments. Figure 10B shows results from MTT assays after treatment of cells with increasing concentrations of NEO214 (0–60 µM) combined with increasing concentrations of bortezomib (0–6 nM). Cell death resulting from combination treatments was much more severe than from individual treatments, in particular when bortezomib concentrations were increased from 2 to 4 and 6 nM. To formally establish whether these effects were synergistic, the data were subjected to statistical analysis based on the Bliss independence model [44]. These calculations showed that combination treatments that included 2 nM bortezomib resulted in additive toxicity, but combination treatments with 4 or 6 nM bortezomib—which represent concentrations that are readily achievable in patients [45]—generated strongly synergistic outcomes (Figure 10C). Comparable synergistic outcomes were achieved with U266 cells as well, emphasizing that the enhanced combination effects are not limited to the RPMI/8226 cell line but might be generally applicable to MM.

## 3. Discussion

The ER stress response/UPR represents a recognized target for therapeutic intervention in cancer, particularly in highly secretory cells such as MM. The clinically used proteasome inhibitor bortezomib appears to exploit this target, at least in part, resulting in proteotoxic cell death [10,11]. Preclinical research efforts from us and others have indicated that it might be possible to achieve superior therapeutic outcomes through the combination of suitable ER stress aggravating agents [6,12]. In this current study, we have explored a novel ERSA combination for MM, based on the use of a newly created, chimeric molecule—NEO214—that turned out to harbor strong ERSA potency.

Intriguingly, covalent conjugation of Rp and POH resulted in a molecule with anticancer potency that was substantially greater not only compared to Rp and POH individually, but also when compared to equimolar combinations of Rp plus POH. In particular, when POH was mixed with Rp for combination treatment, the resultant IC50 was lowered less than two-fold, as compared to individual drug treatments. However, after covalent binding of POH to Rp, as NEO214, the IC50 was lowered by an order of magnitude, as compared to combination treatment with the individual components (Table 1). This large differential was consistently observed in all cell lines investigated and was not limited to cell death assays, but was also evident during the analysis of cellular stress markers. For example, expression of ATF3 and CHOP proteins was readily stimulated by 50 µM NEO214, whereas combinations of Rp plus POH required 1 mM of each compound to achieve a similar effect (Figure 6). Quite clearly, the potency of NEO214 could not be mimicked by a mere mix of its individual components, and thus the activity of the conjugated compound was substantially greater than the sum of its parts.

We also tested NEO214 in normal cells. When added to immortalized, non-tumor cells, the cytotoxic IC50 of NEO214 doubled. More impressively, when applied to truly normal, primary human cells (astrocytes and endothelial cells), we were unable to detect toxic effects of NEO214 even at the highest concentration used (Table 1). These findings are encouraging for the drug’s future clinical testing. In prior clinical trials using oral dosing, both Rp and POH had shown toxicity, primarily dose-limiting GI problems (nausea, vomiting, etc.), which resulted in discontinuation of further development of these oral formulations [26,46]. In the case of POH however, switching from oral to intranasal delivery offered a non-toxic alternative and yielded highly promising results in patients with malignant glioma [27]. In a mouse model of chlorine-induced lung injury, it was demonstrated that intranasal Rp was equally effective as intraperitoneally injected Rp [47], suggesting that—similar to POH—intranasal delivery perhaps could be a less toxic alternative to oral delivery in the case of Rp as well. While NEO214 has not been studied in humans, the above considerations serve as a reminder that future clinical studies of NEO214 should consider its intranasal application, as has been suggested for other chemotherapeutic interventions based on POH-conjugated agents that are under development [48].

What are the cellular targets involved in mediating NEO214-induced cell killing? Prior studies had indicated that stimulation of cyclic AMP signaling via Rp-mediated inhibition of PDE4 can result in growth inhibition of tumor cells of hematopoietic origin, in particular chronic lymphocytic leukemia (CLL), acute lymphocytic leukemia (ALL), and MM cells [49,50,51]. We therefore surmised that PDE4-inhibitory activity of Rp, which was preserved in the chimeric NEO214 molecule (Figure 4), might play a role. However, several points of evidence seem to argue against this model. For instance, although PDE4-inhibitory potency of Rp and NEO214 against purified enzyme is the same (Figure 4A), the cytotoxic potency of NEO214 is 10–20 fold greater than that of Rp (Table 1). Furthermore, if elevated cyclic AMP levels played a key role, one would expect that addition of forskolin would greatly enhance cell death triggered by PDE4 inhibition, as was demonstrated in other studies with ALL, CLL and MM cell lines [49,50]. However, in our cell lines no such enhancement could be observed (Figure 4B). While a convincing contribution of cyclic AMP signaling could not be established, we did however collect substantial evidence supporting a key role for another central cellular process, namely ER stress.

The potential contribution of ER stress was surmised on the basis of our earlier studies with POH, which demonstrated that this monoterpene was able to kill glioblastoma cells at least in part through aggravated ER stress, although fairly high concentrations approaching the millimolar range were required [42]. In the current study, we demonstrate that NEO214 preserved the ER stress-aggravating activity of POH, but at much increased potency. Our data revealed that NEO214 triggered severe ER stress, as indicated by strongly stimulated expression of CHOP protein, the key indicator and central executor of the pro-apoptotic module of the ER stress response system/UPR [2,3]. Although several other components of this cellular stress system cooperate to orchestrate appropriate responses to stress, CHOP has been shown to represent the master regulator that switches the cellular efforts from the initial defensive attempts to subsequent apoptotic execution. In unstressed cells, CHOP levels are generally undetectable or very low, and over-expression of this protein results in cell cycle arrest, which is followed by cell death—unless the elevated expression can be reversed by the defensive efforts of the UPR [3,52]. In our experimental system, we analyzed CHOP levels as a convenient readout for pro-apoptotic ER stress in our experiments. We found that NEO214 resulted in powerful, prolonged stimulation of CHOP expression that was not reversed (Figure 5). CHOP was prominently elevated as early as 4 h after the onset of NEO214 treatment, and even at 48 h after the addition of stimulus, CHOP levels remained very high. In parallel, apoptotic markers (activated caspase 7 and cleaved PARP) began to appear at 4–8 h and continued to increase further over time. Altogether, this mode of response was highly consistent with the dominant function of the pro-apoptotic module of ER stress/UPR and the well-known pivotal role of CHOP [2,53], providing evidence that this mechanism played a decisive role in mediating cell death induced by NEO214.

The key role of ER stress and CHOP was validated with salubrinal and *CHOP*-knockout cells. Salubrinal acts as a phosphatase inhibitor, thereby preventing the dephosphorylation of eukaryotic translation initiation factor 2 alpha (eIF2α) during the ER stress response [54]. Its cytoprotective effects were established in a number of cellular stress conditions induced by various ER stress-aggravating compounds [55,56]. In our experimental system, inclusion of salubrinal exerted cytoprotective effects against NEO214 (Figure 7 and Figure 8). Similarly, significant protection was also provided by knockout of *CHOP* alleles, i.e., in cells where the pro-apoptotic arm of the ER stress response was at least partially amputated (Figure 9). On the other hand, we noted that in both instances, protection was not complete, and cell death still took place at much higher concentrations of NEO214. Therefore, while ER stress and its pro-apoptotic master regulator CHOP clearly are key players in NEO214-induced cell death, it is likely that other cellular components and mechanisms might contribute as well, although perhaps to a lesser extent.

The ERSA principle is based on a model where tumor cells experience chronic ER stress that can be exploited to overload the already engaged response system to exceed the tumor cells’ capacity for adaptation [6]. Due to their secretory phenotype, MM cells might be particularly susceptible to an ERSA-based therapeutic approach, and bortezomib, at least in part, appears to work by this principle [10,12]. Our present study advances this approach further by presenting a novel pharmacological agent, NEO214, that can be applied to synergistically further aggravate the bortezomib-primed system toward optimized tumor cell killing (Figure 10). Should these in vitro synergistic outcomes be validated in the clinic, they would support the use of overall lower drug dosages, which in the patient might result in fewer side effects and an overall higher quality of life during cycles of treatment. While NEO214 has not yet been tested in the clinic, our present in vitro studies do provide support for the above model and encouragement for its further investigation toward clinical development.

## 4. Materials and Methods

### 4.1. Pharmacological Agents

Rolipram, bortezomib, and forskolin were obtained from LC Laboratories (Woburn, MA, USA). Perillyl alcohol, salubrinal, piclamilast, cycloheximide, and DMSO were from Sigma-Aldrich (St. Louis, MO, USA). NEO214 was manufactured by Norac Pharma (Azusa, CA, USA) and was provided by NeOnc Technologies, Inc. (Los Angeles, CA, USA). Chemical purity was 99%, which was confirmed by ^1^H-NMR (300 MHz), mass spectrometry, and microanalysis. The white solid was dissolved in DMSO at 100 mM, and aliquots were stored at –80 °C. NEO214’s IUPAC name is ((S)-4-(prop-1-en-2-yl)cyclohex-1-enyl)methyl 4-(3-(cyclopentyloxy)-4-methoxyphenyl)-2-oxopyrrolidine-1-carboxylate and its CAS number is 1361198-80-2. Molecular formula is C_27_H_35_NO_5_ and molecular weight is 453.57 g/mol.

### 4.2. Cell Lines and Culture

The following human multiple myeloma cell lines were used: RPMI/8226 and U266 (purchased from the American Tissue Culture Collection, ATCC, Manassas, VA, USA), and ARH-77, H929 and Hs-Sultan (kindly provided by Alan Epstein, USC). We also used 8226/Dox40, which is a doxorubicin-resistant subline of the RPMI/8226 cells [57]. All MM cell lines were propagated in RPMI-1640 medium supplemented with 10% fetal bovine serum (FBS), 100 U/mL penicillin, and 0.1 mg/mL streptomycin in a humidified incubator at 37 °C and a 5% CO_2_ atmosphere. As representatives of normal human cells, we used ME16C (mammary gland epithelial cells, immortalized with hTERT; from the ATCC), primary cerebral cortex astrocytes (ScienCell, Carlsbad, CA, USA), and primary brain endothelial cells (BEC), which were isolated and characterized as described previously [58]. ME16C cells were cultured in DMEM supplemented as above, whereas astrocytes were grown in astrocyte medium. BEC were cultured as described earlier [58]. Wild type mouse embryo fibroblasts (MEF wt) and CHOP-knockout derivatives (both from the ATCC) were cultured in DMEM supplemented as above, with additional 0.1 mM non-essential amino acids. All cell culture reagents were provided by the Cell Culture Core Lab of the USC/Norris Comprehensive Cancer Center and prepared with raw materials from Cellgro/MediaTech (Manassas, VA, USA). FBS was obtained from Omega Scientific (Tarzana, CA, USA) and from X&Y Cell Culture (Kansas City, MO, USA).

### 4.3. MTT Assay

Methylthiazoletetrazolium (MTT) assays were performed as described earlier [59]. Briefly, cells were seeded into 96-well plates at 2–5 × 10^4^ cells per well and exposed to drug treatment (or solvent alone) for 24, 48, or 72 h. In individual experiments, each treatment condition was set up in triplicate, and each experiment was repeated several times independently.

### 4.4. Immunoblots

Total cell lysates were analyzed by Western blot analysis as described earlier [60]. Antibodies were obtained from Cell Signaling Technology (Danvers, MA, USA) and Santa Cruz Biotechnology (Santa Cruz, CA, USA). All antibodies were used according to the manufacturers’ recommendations. All immunoblots were repeated at least once to confirm the results.

### 4.5. Determination of Phosphodiesterase Activity

The inhibitory impact of different compounds on PDE4 enzymatic activity was measured with the use of a PDE4A Assay Kit purchased from BPS Bioscience (San Diego, CA, USA). Following the manufacturer’s instructions, purified PDE4A1A recombinant protein was incubated with or without the compound to be tested, in the presence of fluorescently labeled cyclic AMP. After 1 hour at room temperature, a binding agent was added to produce a change in fluorescent polarization that was measured using an Infinite M1000 microplate reader (Tecan, San Jose, CA, USA).

### 4.6. Calculation of Drug Combination Effects

Cells were treated with increasing concentrations of individual drugs, as well as their combinations, and resulting cell toxicity was determined by MTT assay. Drug combination efficacy was evaluated with the Bliss independence model [44], which focuses on treatment effect enhancement. This method compares the observed combination response (Y_O_) with the predicted combination response (Y_P_). Typically, the combination effect is declared synergistic if Y_O_ > Y_P_, additive if Y_O_ = Y_P_, and antagonistic if Y_O_ < Y_P_.

### 4.7. Other Statistical Analysis

All parametric data were analyzed using the Student *t*-test to calculate the significance values. A probability value *p* < 0.05 was considered statistically significant.

## Figures and Tables

**Table 1:** Drug sensitivities of various human cell types.

Cells	Cell Type	NEO214	Rp	POH	Rp + POH	*p*-Value *
RPMI/8226	MM	50	551	>1000	482	<0.0001
8228/Dox40	MM	59	n.d.	n.d.	n.d.	<0.0001
U266	MM	56	>1000	>1000	606	<0.0001
ARH-77	MM	52	>1000	>1000	783	<0.0001
H929	MM	50	578	>1000	486	<0.0001
Hs-Sultan	MM	55	>1000	>1000	789	<0.0001
ME16C	normal breast	118 **	n.d.	n.d.	n.d.	n.d.
Astrocytes	normal brain	>>200 ***	n.d.	n.d.	n.d.	n.d.
BEC	brain endothelial	>>200 ***	n.d.	n.d.	n.d.	n.d.

Shown are average IC50 values in µM (as determined by MTT assay). *n* ≥ 3. * *p*-Value: statistical difference between treatment with NEO214 and any of the three other conditions (Rp, POH, Rp + POH). ** *p* < 0.01 for difference between MM cancer cells and immortalized normal breast epithelial cells. *** 200 µM was the highest concentration tested and there was no detectable toxicity in these normal primary cells. n.d.: not determined. ME16C, mammary gland epithelial cells; BEC, brain endothelial cells.

## Abbreviations

ATF3	Activating transcription factor 3
BZM	Bortezomib
CHOP	CCAAT/enhancer-binding protein homologous protein (also called GADD153)
ER	Endoplasmic reticulum
ERSA	Endoplasmic reticulum stress aggravator
FSK	Forskolin
γH2AX	Phosphorylated form of histone variant H2AX protein
GI	Gastrointestinal
IC50	Inhibitory concentration at 50% effect
MM	Multiple myeloma
NEO214	Perillyl alcohol conjugated to rolipram
PARP	Poly [ADP-ribose] polymerase
POH	Perillyl alcohol
Rp	Rolipram
